# Dental Occlusion Characteristics for Treatment Decision-Making Regarding Surgery-First Approach in Orthodontics

**DOI:** 10.3390/jcm12186029

**Published:** 2023-09-18

**Authors:** Ying-Chen Chen, Carol Yi-Hsuan Chen, Min-Chi Chen, Ellen Wen-Ching Ko, Cheng-Hui Lin

**Affiliations:** 1 Graduate Institute of Dental and Craniofacial Science, Chang Gung University, Taoyuan 333, Taiwan; flag30276@gmail.com; 2 Department of Craniofacial Orthodontics, Chang Gung Memorial Hospital, Taoyuan 333, Taiwan; acantehr@gmail.com; 3 Department of Craniofacial Orthodontics, Chang Gung Memorial Hospital, Taipei 105, Taiwan; 4 Department of Public Health and Biostatistics Consulting Center, School of Medicine, Chang Gung University, Taoyuan 333, Taiwan; mcc@mail.cgu.edu.tw; 5 Craniofacial Research Center, Chang Gung Memorial Hospital, Linkuo, Taoyuan 333, Taiwan; 3344@cgmh.org.tw; 6 Department of Plastic and Reconstructive Surgery, Chang Gung Memorial Hospital, Taoyuan 333, Taiwan

**Keywords:** surgery-first approach (SFA), mandibular prognathism, digital dental model, lateral cephalogram, surgical orthodontics

## Abstract

The surgery-first approach (SFA) is conducted to decrease the difficulty and duration of orthodontic treatment by correcting the skeletal discrepancy at the initial stage of treatment. However, the indication of the SFA has not been well defined yet. This study explored the dental occlusion characteristics for treatment decision-making regarding the SFA. A total of 200 skeletal Class III patients were consecutively collected and divided into two groups: the orthodontic-first approach (OFA) group and the SFA group. The pretreatment digital dental models and lateral cephalograms were measured. Logistic regression was completed and receiver operating characteristic (ROC) curves were obtained to predict the probability of the SFA. Results showed that the ROC model with L1-MP, upper and lower arch length discrepancy, overbite, and asymmetric tooth number as influencing factors revealed that the sensitivity and specificity for determining SFA were 83.0% and 65.0%, respectively; the accuracy of prediction was 75.0%. In conclusion, our findings indicate that the six measurements from digital dental models and lateral cephalograms can be effectively applied in treatment decision-making for the SFA with satisfactory accuracy.

## 1. Introduction

The conventional surgical–orthodontic treatment for patients with Class III deformities is typically performed as a three-stage approach: presurgical orthodontic treatment, orthognathic surgery, and postoperative treatment [1,2,3]. Although this approach provides satisfactory results, management of dental decompensation during presurgical orthodontic movement requires a long treatment duration that may upset the equilibrium of the muscular environment and result in a worsened facial profile and masticatory function [4]. Hence, the surgery-first approach (SFA), which reduces the need for presurgical orthodontic treatment, is gaining attraction [4,5]. Orthognathic surgery induces a regional acceleratory phenomenon, which decreases the difficulty and duration of orthodontic treatment [6,7,8]. Moreover, early correction of skeletal discrepancies can improve quality of life [9]. Advancements in surgical techniques, such as the use of rigid fixation and the refinement of segmental osteotomy procedures, coupled with the integration of computer-assisted simulations, have expanded the applicability of the SFA to a broader range of cases [5,10,11]. Furthermore, both approaches show no significant difference in terms of surgical stability [4,12].

The criteria for the SFA include the following parameters: alignment of dentition, inclination of anterior teeth, curve of Spee, and transverse discrepancy [4,6,13,14]. Regarding alignment, most studies have included cases with only mild crowding (<5 mm) for investigations of the SFA [12,15,16,17,18]. Choi et al. suggested that patients with crowding or spacing might require presurgical orthodontic consolidation of dental spaces [15]. Huang et al. indicated that more experienced surgeons and orthodontists may perform premolar extraction during orthognathic surgery in cases of severe crowding [17]. Regarding proclined maxillary incisors, a study indicated that the SFA can lead to similar surgical stability, but cases involving upper incisor inclination (U1-SN) > 122° requiring extraction were excluded [18]. However, some studies have attempted premolar extraction during surgery and corrected such inclinations later using the SFA. Their results have revealed similar inclination alterations when premolar extraction was performed before or during surgery [16,17]. The aforementioned findings indicate that reports regarding the selection criteria related to incisor inclination remain inconsistent. Ko et al. identified the curve of Spee as a factor causing mandibular relapse and recommended that a deep curve of Spee be leveled before orthognathic surgery [19]. For transverse discrepancy, surgical-assisted rapid palatal expansion before orthognathic surgery could diminish the discrepancy [9,20]. However, patients would suffer from two surgical procedures. With the evolution of surgical techniques, a combination of rigid fixation and segmental osteotomies to expand the dental arch in the same surgery can simplify the treatment procedure [13,19,21,22].

Factors related to skeletal discrepancy have also been proposed as indicators of whether the SFA should be employed. Hernández-Alfaro et al. recommended that patients who present severe facial asymmetry with dental compensation should be excluded from consideration for the SFA [9]. However, with the use of computerized surgical simulation and advancements in surgical techniques, the accuracy of facial asymmetry correction through the SFA has been increasing [23,24]. Moreover, in patients with obstructive sleep apnea who require orthognathic surgery, correcting the skeletal structure was reported to require prioritization to prevent them from experiencing worsening respiratory function; therefore, these patients should be considered as candidates for the SFA [9]. Additionally, several articles have suggested using surgical simulation and digital models for the selection of candidates for the SFA or the orthodontic-first approach (OFA) [25,26].

Thus, although the SFA was an aesthetic/skeletal-first concept, the factors determining patient selection for this approach have mainly been dental characteristics. The optimal surgical occlusion design for the surgery-first approach is a “treatable malocclusion” that does not interfere with surgical movement [15,17]. Unlike conventional surgical orthodontics, deep overbites or posterior open bites can typically be managed using the SFA [17,27]. Such occlusion can be simply corrected during postsurgical orthodontic treatment, and this facilitates dental decompensation and prevents anterior open bites. However, the dental interference that causes unstable surgical occlusion might contribute to mandibular counterclockwise rotation when settling the occlusion in post-operative orthodontics. Moreover, unavoidable interference may limit the amount of jaw repositioning [15]. Nevertheless, several studies have revealed that the SFA and OFA have comparable stabilities [12,18]. Therefore, the establishment of selection criteria based on surgical occlusion design might enhance postoperative stability and predictability [12,28,29].

As a result, the evaluation of dental occlusion plays a crucial role in the selection of the Surgery First Approach (SFA) or the Orthodontic First Approach (OFA). Digital models offer several advantages over traditional plaster models, including cost-effectiveness, time efficiency, and storage convenience. Furthermore, digital models are highly reliable and exhibit exceptional accuracy, positioning them as the modern gold standard in orthodontic practice [30,31]. In this study, we utilized digital dental models to assess dental occlusion characteristics.

At the Chang Gung Craniofacial Center in Taipei, Taiwan, the SFA has been consistently applied for an extended duration. The proficient surgeons have developed several refinements to surgical techniques, enabling them to effectively achieve sagittal, vertical, and transverse dental decompensation through segmental osteotomy, thereby broadening the indications for the SFA [19]. Moreover, the use of computer-aided simulation and three-dimensional printing of surgical splints has enhanced surgical movement accuracy. In the present study, we identified the dental occlusion characteristics that can aid in treatment decision-making regarding SFA and OFA selection for Class III deformities. The aim of this study was to develop a scoring system to determine the necessity of presurgical orthodontic treatment based on a dental model and the lateral cephalogram variables of patients with Class III malocclusion.

## 2. Material and Methods

This retrospective cohort study examined a series of consecutive patients who underwent surgical–orthodontic treatment. The inclusion criteria were (1) Taiwanese adult aged ≥20 years, (2) having skeletal Class III malocclusion with an ANB angle ≤ 0, (3) having undergone two-jaw surgery, (4) having ≤2 missing teeth per quadrant (third molars are not included), (5) having completed postsurgical orthodontic treatment, and (6) having pretreatment cephalometric radiographs and digital dental models. Patients with craniofacial anomalies, facial trauma history, and incomplete diagnostic records were excluded.

This study included 200 patients who were divided into two groups according to their treatment modality: the OFA group (41 men and 59 women who received presurgical orthodontic treatment) and the SFA group (39 men and 61 women who received orthognathic surgery before any orthodontic treatment). As the design in surgical occlusion necessitates careful consideration, the responsibility of selecting appropriate cases for SFA falls upon an experienced orthodontist. The selection of the OFA or SFA was based on the initial model manipulation and surgical occlusion management. The occlusion with interference and the inability to set up proper surgical occlusion were categorized into the OFA group. The clinical reasons for selecting the OFA were documented in clinical charts. This study was approved by the Institutional Review Board and Medical Ethics Committee of Chang Gung Memorial Hospital (IRB No. 201901921B0) and followed the guidelines of the Declaration of Helsinki.

### 2.1. Preparation and Orientation of Digital Models

To create digital models, pretreatment plaster casts were scanned using an extraoral scanner (R700, 3Shape, Copenhagen, Denmark), and the Standard Tessellation Language file was transferred to OrthoAnalyzer software (3Shape) for digital model analysis.

**Figure 1:**
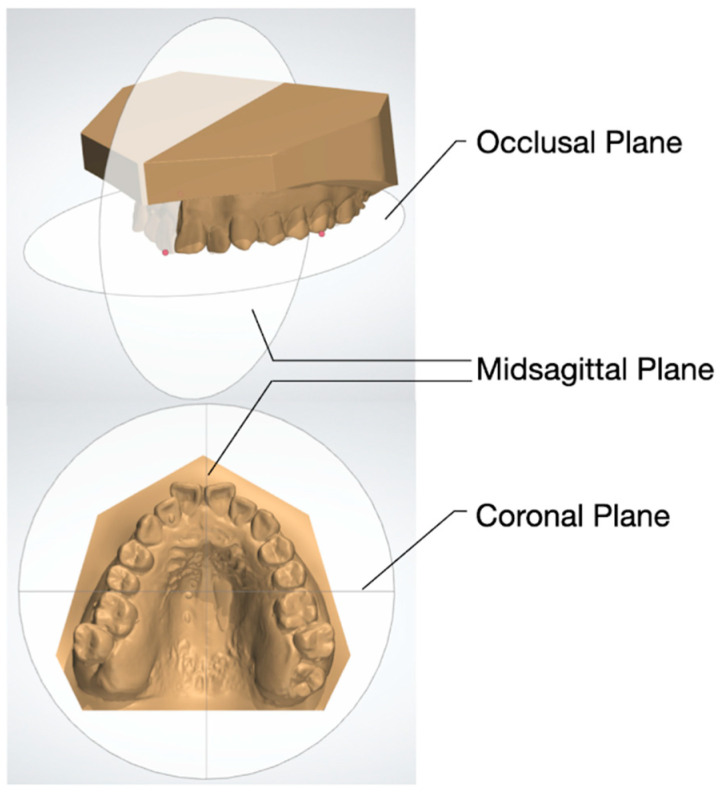
Three-dimensional planes for digital model orientation and measurement.

The following three-dimensional planes were used to standardize the orientation and improve the accuracy of the dental model measurement: (1) the occlusal plane, passing through the mesiobuccal cusp of the bilateral first molars and the most extruded point on the incisal edge of the central incisors; (2) the midsagittal plane, passing through the center of the incisive papilla and midpalatine raphe, perpendicular to the occlusal plane; and (3) the coronal plane, separating the anterior and posterior regions and perpendicular to the occlusal plane and midsagittal plane (Figure 1).

### 2.2. Variables of Dental Model Measurements

#### 2.2.1. Overbite and Overjet

On the plane parallel to the midsagittal plane passing through the middle of the central incisors, the patients’ overbites and overjets were measured, and the average values were used.

#### 2.2.2. Arch Length Discrepancy (ALD)

ALD was defined as the absolute space required minus the dental arch length, including UALD and LALD (upper and lower arches). The space required was calculated as the sum of the mesiodistal width of the premolars and anterior teeth. The dental arch length was measured using the ideal arch form on the virtual occlusal plane, in which a curved arch length was measured relative to the apical base.

#### 2.2.3. Intercanine Width (ICW) and Intermolar Width (IMW) Discrepancy

The transverse discrepancy included the ICW discrepancy and IMW discrepancy, in which the maxillary value minus the mandibular value was obtained.

#### 2.2.4. Arch Form Asymmetry Index (AI)

The difference in the distance between the midsagittal plane and the bilateral cusp tip of the canines, premolars, and molars was measured. The square root of the squared sum of the three numbers was calculated as the AI to generate an absolute number.

#### 2.2.5. Asymmetric Tooth Number (ATN)

The tooth number from the central incisor to the first molar was recorded bilaterally. It was then categorized into dichotomized variables as “same” or “different”.

#### 2.2.6. Curve of Spee

**Figure 2:**
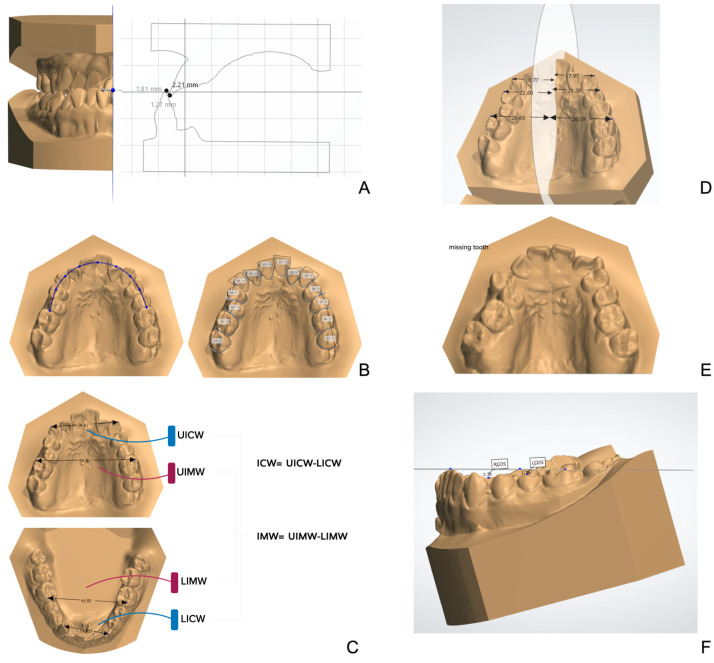
Measurements of dental characteristics with digital dental models. (**A**) OB and OJ measurement on the plane parallel to midsagittal plane pass through the middle of central incisors. (**B**) Arch length discrepancy was defined as the absolute number of spaces required minus dental arch length. Dental arch length was measured using the ideal arch method on the occlusal plane, with the curve length measured relative to the apical base. (**C**) Intercanine width (ICW) and intermolar width (IMW) discrepancy. (**D**) Asymmetry index (AI) of the arch form. (**E**) Asymmetric tooth number (ATN). (**F**) Curve of Spee (COS), the depth of the COS was measured as the perpendicular distance between the deepest cusp tip to the plane touching the incisal edges of the central incisors and the distal cusp tips of the first molar in the lower arch.

The depth of the curve of Spee was measured as the perpendicular distance between the deepest cusp tip to the plane touching the incisal edges of the central incisors and the distal cusp tips of the first molar in the lower arch. The average value was recorded (Figure 2).

### 2.3. Variables of Cephalometric Measurements

**Figure 3:**
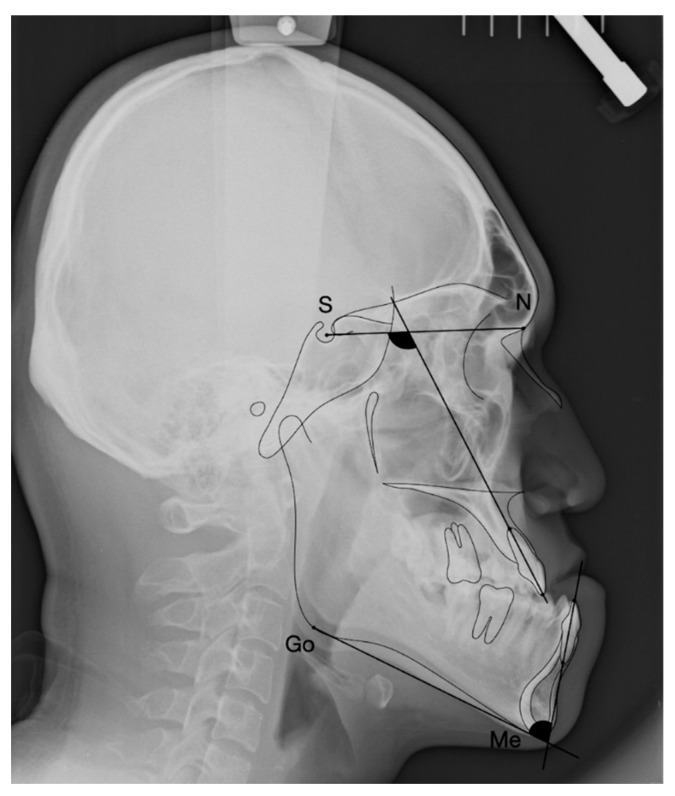
The measurements of incisor inclination of the maxilla (SN-U1) and the mandible (MP-L1) on the lateral cephalograms. S, sella; N, nasion.

Cephalometric measurements were obtained using digital lateral cephalograms in Centricity Enterprise (GE Medical System, Parramatta, Australia), and the incisor inclination of the maxilla (SN-U1) and mandible (MP-L1) was measured (Figure 3).

### 2.4. Statistical Analysis

Between-group differences were compared using the independent *t*-test. Logistic regression analyses were performed to identify the predictors of candidacy for the SFA. Backward stepwise multiple logistic regression was applied to develop a prediction sequence for the variables. Receiver operator characteristic (ROC) analysis was performed to identify the cutoff point of each variable to determine its distinguishing ability. The scoring system was developed according to the cutoff points and prediction ability sequence. Cumulative ranked scores were analyzed with ROC curves and univariate logistic regression to determine the final prediction ability and accuracy of the model by comparing the estimated results with actual observations. All statistical analyses were conducted using SPSS v22.0 (IBM, Chicago, IL, USA).

All measurements were conducted by a single examiner (Y.C.C.). Ten digital casts were randomly selected to conduct a reexamination after an interval of 2 weeks. The validation of measurement consistency was assessed. A paired *t*-test was used for systematic errors. The interclass correlation coefficient (ICC) test was used to test reproducibility.

## 3. Results

For the demographic characteristics, this study included 100 patients in the SFA group (34 men, 66 women; mean age 20.4 years) and 100 patients in the OFA group (37 men, 63 women; mean age 22.4 years). The sex and age distributions were not significantly different between the two groups. The mean period of presurgical orthodontic treatment was 6.9 months, during which alignment, leveling, partial incisor inclination correction, and anterior arch coordination were performed, depending on the objective of the case (Table 1). The ANB angle was −3.3° and −3.6° in the SFA and OFA groups, respectively, indicating no significant difference. For the measurement accuracy, the paired *t*-test results indicated no significant difference between the two sets of measurements. The ICC test indicated excellent measurement reproducibility (0.96–0.99).

**Figure 4:**
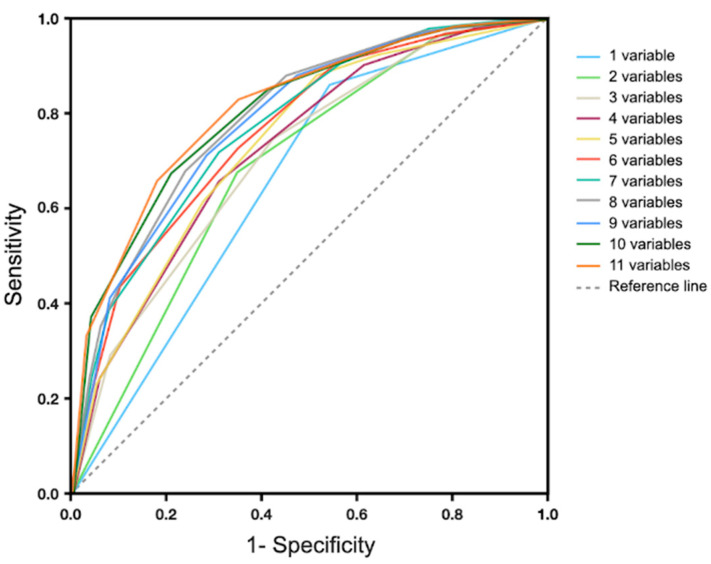
The bar chart indicates the reasons for OFA that were obtained from clinical chart review.

Based on dental model manipulation for the surgical occlusion setup, the necessity of presurgical orthodontic treatment was determined for each patient. The indicators for selecting the OFA are listed in Table 2 and Figure 4, with some cases having more than one indicator. The most common indicator was severe dental crowding, followed by dental interference, incisor proclination, dental space, narrow arch forms, uncoordinated dental midline within dental arch, regaining space for osteotomy, and deep curve of Spee.

The OFA group exhibited more upright lower incisor inclination than the SFA group did (*p* < 0.05). The upper incisor inclination did not significantly differ between the two groups (*p* > 0.05). However, compared with the SFA group, the OFA group exhibited more severe crowding and spacing in both the upper and lower arches and significantly deeper overbite and curve of Spee (all *p* < 0.01). For the arch form and tooth number symmetry, the OFA group exhibited a greater AI and a higher prevalence of asymmetric tooth number in bilateral arch than the SFA group did (*p* < 0.05) (Table 3 and Table 4).

To develop a scoring system, this study first used the ROC curves to determine cutoff points by using the maximum sum of the sensitivity and specificity for each variable (Table 5). The parameters were subsequently categorized into scores of 1 or 0 based on a defined cutoff point. In terms of clinical interpretation, occlusion characteristics indicative of a propensity for SFA were assigned a score of 1.

**Figure 5:**
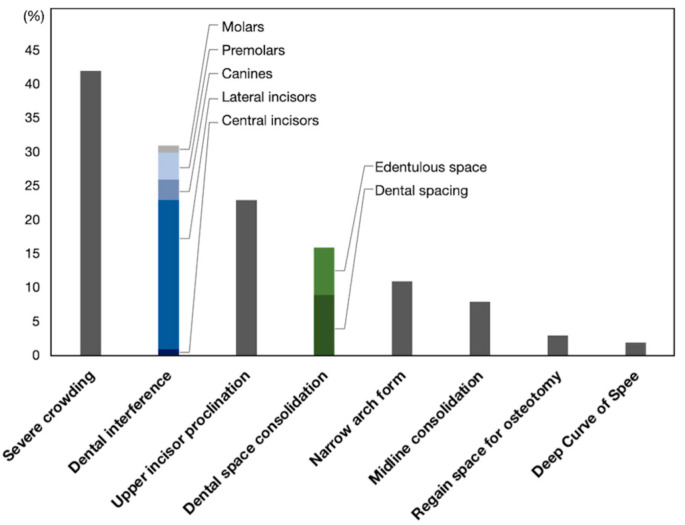
Area under the curve (AUC) in the dichotomized scoring system. The AUC increases as more variables are included. The results indicate the six-variable scoring system was the most efficient with the largest AUC and best diagnostic accuracy.

The study employed logistic regression to establish the order of variables by predictability, with UALD, OB, MP-L1, LALD, ATN, SN-U1, curve of Spee, OJ, AI, IMW, and ICW in descending order. Scores were cumulatively aggregated following this sequence. The dichotomized scoring system showed a larger AUC than the original parameters, enhancing discriminative power. The study aimed for an effective diagnostic model by identifying the most accurate scoring system instead of using all variables. It found that a six-variable system (UALD, OB, MP-L1, LALD, ATN, SN-U1) had the highest AUC (0.8) and diagnostic accuracy (75%) (Table 6, Figure 5). Each variable in the system was scored as 0 or 1, yielding a total score range of 0 to 6. Analyzing the cutoff point for grouping, logistic regression was used to calculate prediction accuracy and SFA probability for each score (Table 7). Results showed that a total score of 4 had the best sum of sensitivity (0.83) and specificity (0.65), offering the highest diagnostic accuracy. Scores of 4, 5, and 6 corresponded to probabilities of 58%, 83%, and 94%, respectively, for categorization in the SFA group. In conclusion, the six-variable scoring system effectively identified Class III malocclusion patients suitable for the SFA treatment.

## 4. Discussion

With advancements in digital dentistry, the use of digital dental models for orthodontic analysis is becoming increasingly popular. Compared with those of plaster models, the reproducibility and repeatability of the measurements of digital models were compatible [32,33]. In this study, a three-dimensional plane system was constructed to facilitate digital model assessment. In our previous study, which compared segmental distances with curved ideal arch lengths for space analysis, revealed that the ideal arch form method can characterize the real arch length of the basal bone and the arch length can simply be obtained and measured using digital software [33,34]. Furtermore, every parameter in the present study had excellent reproducibility, with a high ICC value, indicating that digital models can generate reliable and reproducible measurements.

Given that the establishment of surgical occlusion as a treatable malocclusion in SFA is a prerequisite, the responsibility of selecting appropriate cases for SFA falls upon an experienced orthodontist. Additionally, the orthodontist not only formulates the surgical plan but also collaborates with surgeons to incorporate any necessary modifications. This underscores the orthodontist’s comprehensive understanding of the interplay between surgical occlusion and the intended surgical movements. It is important to note, however, that different specialists might approach the selection process differently. For instance, some more seasoned orthodontists may designate all patients as potential candidates for SFA. Nonetheless, achieving predictable outcomes demands a higher degree of surgical technique and experience. In this study, while the cases are divided into two categorical groups for selection purposes, they are ultimately integrated into a scoring system. This scoring system would be invaluable for orthodontists and surgeons alike, irrespective of orthognathic teams’ levels of experience and institutes.

To the best of our knowledge, this study is the first to quantify the occlusion characteristics of patients with Class III malocclusion and to evaluate the probability of variables being candidates for the SFA by using ROC curves. Patients with a score of more than 4 points on our six-variable scoring system are likely to be candidates for the SFA, with a prediction accuracy of 75%. Five of the six variables (UALD, OB, MP-L1, LALD, and ATN) were significantly different between the two study groups. Although SN-U1 is not, the prediction ability, including the sensitivity and specificity of the factor, is measured using the ROC curve rather than the independent *t*-test. An independent *t*-test compares only differences in mean values; on the other hand, the curve of receiver operating characteristic could reveal the grouping probability. This scoring model shows high sensitivity which indicates that it can more precisely select patients for the SFA. The medium level of specificity of the prediction model can be compensated through modification: in borderline cases, that is, cases with a score of 4 points, consideration of other parameters, such as dental transverse discrepancy, curve of Spee, asymmetric arch form, or directly surgical occlusion manipulation might be applied for selection of treatment modalities.

Our previous study compared occlusion parameters by using digital occlusal analysis (T-Scan) on patients for whom the SFA and OFA were applied. Sharing the same case selection philosophy as this study, the results of the study indicated that all occlusion parameters, including force discrepancy, occlusal time, maximum forced percentage, and number of teeth in contact, after surgery were similar in the SFA and OFA groups [35]. The study showed that with this selection principle, the SFA group and the OFA group could have similar treatment outcomes regarding dental occlusion.

Skeletal Class III patients are typically associated with maxillary deficiency and therefore present with upper dental crowding and labially proclined upper incisors [36,37]. The most decisive variable among the occlusion characteristics is UALD. The arch length discrepancies in our study included dental crowding and dental spacing. Liou et al. and Park et al. excluded patients with severe crowding (>5 mm) and extraction requirements for SFA [6,18]. For patients with space deficiency, interference from dental malignment can influence the surgical occlusion setup and might cause unstable surgical jaw positioning—in particular, palatoversion of the lateral incisors. In these patients, anterior surgical occlusion cannot achieve proper overbite. Although Huang et al. suggested extracting premolars in patients with severe crowding during orthognathic surgery, this procedure requires sophisticated surgical techniques and precise tooth movement prediction, which might be difficult for inexperienced orthodontists and surgeons [17]. However, a short period of leveling and alignment to relieve major interference can improve surgical occlusion setup. Aside from dental interference, patients with crowded dentition, significant spacing or ATN might present with dental midline deviations within the arch. Presurgical orthodontic alignment might assist in identifying the relative center position between the dental arch and the aligned jaw bones and prevent surgeons from being misguided of the dental midline [38,39].

For correction of upper incisor inclination or compensation, there are several treatment options. For the severe upper incisor proclination, upper premolar extraction followed by anterior teeth retraction is common in the OFA group for decompensation [6,29]. In addition, clockwise rotation of the maxillomandibular complex (MMC), or segmental osteotomy accompanied with anterior segment clockwise rotation, can also be performed for inclination correction of upper incisors during surgery. The limited amount of posterior maxillary impaction (in MMC clockwise rotation) and the vertical gingival discrepancy between maxillary segments (in segment rotation) should be considered for the limitation to correct the upper incisor inclination. A rotation of more than 10 degrees would cause a marked vertical step between the canines and the second premolars in segmental osteotomies to close the first premolar extraction space and bony gap [36,40]. A study suggested intraoperative upper premolar extraction for cases with severely proclined incisors. For this procedure, clinical experience of prediction in lip profile changes and post-operative orthodontic finishing movement is required for setting up large overjet and Class II surgical occlusion during surgery [17]. However, some patients cannot tolerate the dramatic profile change from mandibular prognathism to protrusive maxilla, even though it is temporary. According to the results of this study, an upper incisor inclination (U1-SN) of >120.8° might undergo upper premolar extraction and incisor retraction in presurgical preparation until the remaining inclination can be further rotated by segmental osteotomy or MMC clockwise rotation.

The results of this study show that patients with shallow overbites (OB ≤ 2 mm) and retroclined lower incisor angles (MP-L1 ≤ 76.1°) had a higher tendency to undergo the SFA. These characteristics typically show more in Class III high mandibular plane angle patients. Uribe et al. indicates that Class III malocclusion with anterior open bite usually has mild crowding and less dental compensation; thus, they are good candidates for SFA [23]. In other words, patients with mandibular prognathism and overclosure tend to have underdeveloped maxilla and show more severe crowding or deep overbite which would require limited presurgical orthodontic treatment. In patients with an accentuated curve of Spee, leveling with continuous archwire during the postoperative period causes mandible counterclockwise rotation through relative lower incisor intrusion. For the sake of surgical stability, leveling the curve of Spee before surgery or surgical overcorrection is necessary if SFA is selected [36,41].

The small number of patients with facial asymmetry indicates that asymmetric arch form is not a determining factor for candidacy for the OFA. The asymmetric arch form might be due to dental compensation in facial asymmetry. The side shift of mandibular surgical movement is restricted by the palatal inclination of the maxillary arch on the non-deviated side. In patients with severe dental compensation, presurgical arch expansion through posterior teeth decompensation and increasing upper intercanine width (ICW) can aid in the surgical occlusion setup, enabling a wider range of mandibular side shifting during the surgical correction of facial asymmetry. Moreover, for cases presenting Class I occlusion with a complete palatal crossbite in hand articulation, presurgical decompensation can be considered to prevent postsurgical interference. Conversely, maxillary basal bone asymmetry can be addressed through segmental osteotomy to expand the dental arch in cases of skeletal facial asymmetry in SFA. 

ICW and IMW were the least decisive factors according to the scoring system. Previous studies reported that the ICW of Class III was similar to that of patients with Class I malocclusion, and the IMW of Class III was smaller than Class I subjects [42,43]. However, some patients with Class III malocclusions presenting narrow upper ICW might still require presurgical ICW expansion for proper surgical occlusion setup. Employing segmental osteotomies to adjust the arch width in cases with major skeletal transverse discrepancy could reduce the presurgical time of OFA or even transition such cases into SFA [44]. 

Based on the results of the study, all the presurgical orthodontic treatments were aimed at (1) eliminating major dental interference, (2) consolidating dental space and the coordinate dental midline within the arch, (3) facilitating major arch form alteration by preparing space and solving future problems for segmental osteotomies, and (4) enhancing surgical stability if major closing movement of mandible is predicted such as a deep curve of Spee in skeletal Class III deformities. On the other hand, presurgical orthodontic treatment should not aim for full dental decompensation and full arch coordination with a lengthy period of presurgical orthodontics. Only a short period of orthodontic treatment of about six months, eliminating the main interference for surgical occlusion setup, can be achieved. Other than the aforementioned conditions, SFA can be conducted with applaudable and stable outcomes. According to the result of this study, the scoring system can be a screening tool for decision making of the surgical–orthodontic treatment modalities: SFA or OFA, especially for beginners in SFA. 

The study was retrospectively designed, a setup that tends to introduce more limitations to the findings. In order to mitigate this bias, it would be contemplating the possibility of devising a multicenter prospective randomized trial in the future.

## 5. Conclusions

- Patients who require presurgical orthodontic treatment tend to have more upright lower incisor inclination, more crowded or spaced dentition in both arches, a deep curve of Spee and overbite, and an asymmetric arch form in Class III surgical orthodontics.
- For optimal discriminant effectiveness between SFA and OFA, six variables were selected to predict the probability of SFA candidacy with an accuracy of 75%, including UALD ≤ 4.1 mm, OB ≤ 2.0 mm, MP-L1 ≤ 76.1 mm, LALD ≤ 1.4 mm, no ATN, and SN-U1 ≤ 120.8°.
- In the scoring system, a score of 4 provided a favorable prediction of the probability of SFA candidacy, with a sensitivity of 83% and specificity of 67%.
- In borderline cases with a score of 4 points, other parameters such as dental arch transverse discrepancy, asymmetric arch form, curve of Spee, or computed surgical simulation may be considered for the decision of treatment modalities.
- The scoring system could simplify the decision-making procedure for SFA and OFA.

## Figures and Tables

**Table 1:** Descriptive data of the patients.

		OFA Group *n* = 100	SFA Group *n* = 100	*p*-Value
Age (Mean ± SD)		22.40 ± 4.87	20.44 ± 3.25	0.44
Gender	Male	37 (37%)	34 (34%)	
	Female	63 (63%)	66 (66%)	
Presurgical orthodontic treatment period (month)		7.14	0	

**Table 2:** Reasons for presurgical orthodontic treatment, obtained from chart review.

Reasons for Presurgical Orthodontics (Total *n* = 100)	*n*
Severe crowding	42 (42%)
Dental interference	31 (31%)
– Central incisors	1
– Lateral incisors	22
– Canines	3
– Premolars	4
– Molars	1
Upper incisor proclination	23 (23%)
Dental space consolidation	16 (16%)
– Spacing	9
– Edentulous space	7
Midline consolidation	8 (8%)
Narrow Arch form	11 (11%)
Regain space for osteotomy	3 (3%)
Deep Curve of Spee	2 (2%)

**Table 3:** Comparison of measurements on digital dental models between the SFA and OFA.

	SFA *n =* 100		OFA *n =* 100		
	Mean	SD	Mean	SD	*p*-Value
ANB	−3.3	2.5	−3.6	2.7	0.73
SN-U1	111	8.45	112.20	13.79	0.51
MP-L1	79.13	9.02	81.80	9.19	0.04 ^†^
UALD	2.16	1.94	4.71	4.39	<0.001 ^‡^
LALD	3.13	2.74	4.55	3.44	0.001 ^‡^
ICW	8.11	2.54	8.18	2.96	0.86
IMW	7.52	4.05	8.38	6.45	0.26
OB	0.13	2.43	1.71	3.01	<0.001 ^‡^
OJ	−1.68	2.73	−2.09	2.96	0.32
COS	0.72	0.79	1.11	1.00	0.002 ^‡^
AI	1.41	0.82	1.74	1.05	0.01 ^†^

Independent *t*-test. U1, SN-U1; L1, L1-MP; UALD, upper arch length deficiency; LALD, lower arch length deficiency; ICW, intercanine width; IMW, intermolar width; OB, overbite; OJ, overjet; COS, curve of Spee; AI, arch form asymmetry index. † *p* < 0.05, ‡ *p* < 0.01.

**Table 4:** Comparison of asymmetric tooth number between the SFA and OFA.

		SFA *n* = 100	OFA *n* = 100	
		Prevalence	Prevalence	*p*-value
ATN	Yes	28.6%	71.4%	0.02 ^†^
	No	53.5%	46.5%	

Chi-square test. ATN, asymmetric tooth number. † *p* < 0.05.

**Table 5:** The area under the curve (AUC) of all measurements.

Variable	AUC	Cutoff Point	Score 1	Score 0	Sensitivity	Specificity	Youden Index
UALD	0.685	4.12	<4.0	≥4.0	0.85	0.46	0.31
OB	0.644	2.0475	<2.0	≥2.0	0.81	0.4	0.21
LALD	0.632	1.355	<1.4	≥1.4	0.38	0.85	0.23
COS	0.619	1.13	<1.1	≥1.1	0.76	0.47	0.23
AI	0.602	22.2	<22.2	≥22.2	0.89	0.28	0.16
ATN	0.56	-	NO	YES	-	-	-
L1	0.582	76.1	<76.1	≥76.1	0.44	0.76	0.2
U1	0.545	120.75	<120.8	≥120.8	0.89	0.24	0.13
OJ	0.528	−0.79	>−0.8	≤−0.8	0.37	0.75	0.12
ICW	0.473	5.995	>6	≤6	0.85	0.27	0.12
IMW	0.447	3.94	>3.9	≤3.9	0.87	0.19	0.06

A higher AUC indicated stronger prediction power of the variable. The corresponding cutoff point with the maximum sum of the sensitivity and specificity values was determined for each variable. The values of each variable with a tendency for orthognathic surgery were scored as 1, and the others were scored as 0.

**Table 6:** Scoring systems of prediction for SFA in the cumulative top-ranked measurements (cumulative scores).

Number of Cumulated Top–Ranked Variables	Variable	AUC	Accuracy
1 variable	UALD	0.67	67
2 variables	above + OB	0.70	66.5
3 variables	above + L1	0.74	69
4 variables	above + LALD	0.76	69
5 variables	above + ATN	0.78	71
6 variables	above + U1	0.80	75
7 variables	above + COS	0.80	74.5
8 variables	above + OJ	0.79	73.5
9 variables	above + AI	0.79	71.5
10 variables	above + IMW	0.80	73
11 variables	above + ICW	0.81	74.5

**Table 7:** Identification of the cutoff point of the scoring system based on three dichotomized variables.

Number of Dichotomized Variables	Probability of SFA	Sensitivity	Specificity	Sensitivity+ Specificity	True+	True−	False+	False−	Accuracy
6	0.94	0.05	0.99	1.04	5	99	1	95	52
5	0.83	0.45	0.9	1.35	45	90	10	55	67.5
4	0.58	0.83	0.67	1.5	83	67	33	17	75
3	0.29	0.97	0.31	1.28	97	31	69	3	64
2	0.11	1	0.03	1.03	100	3	97	0	51.5
1	0.04	1	0	1	100	0	100	0	50
0	0	1	0	1	5	99	1	95	52

## Data Availability

Please contact the corresponding author if someone wants to request the data for this study.
